# Surface States of (100) O-Terminated Diamond: Towards Other 1 × 1:O Reconstruction Models

**DOI:** 10.3390/nano10061193

**Published:** 2020-06-18

**Authors:** Gonzalo Alba, M. Pilar Villar, Rodrigo Alcántara, Javier Navas, Daniel Araujo

**Affiliations:** 1 Departamento de Ciencia de los Materiales e IM y QI, Facultad de Ciencias, Universidad de Cádiz, 11510 Puerto Real (Cádiz), Spain; pilar.villar@uca.es (M.P.V.); daniel.araujo@uca.es (D.A.); 2 Departamento de Química-Física, Facultad de Ciencias, Universidad de Cádiz, 11510 Puerto Real (Cádiz), Spain; rodrigo.alcantara@uca.es (R.A.); Javier.navas@uca.es (J.N.)

**Keywords:** O-terminated diamond, H-terminated diamond, diamond surface reconstruction, angle-resolved XPS

## Abstract

Diamond surface properties show a strong dependence on its chemical termination. Hydrogen-terminated and oxygen-terminated diamonds are the most studied terminations with many applications in the electronic and bioelectronic device field. One of the main techniques for the characterization of diamond surface terminations is X-ray photoelectron spectroscopy (XPS). In this sense, the use of angle-resolved XPS (ARXPS) experiments allows obtaining depth-dependent information used here to evidence (100)-O-terminated diamond surface atomic configuration when fabricated by acid treatment. The results were used to compare the chemistry changes occurring during the oxidation process using a sublayer XPS intensity model. The formation of non-diamond carbon phases at the subsurface and higher oxygen contents were shown to result from the oxygenation treatment. A new (100) 1 × 1:O surface reconstruction model is proposed to explain the XPS quantification results of O-terminated diamond.

## 1. Introduction

Diamond exhibits very interesting bulk properties, such as a wide band gap of 5.5 eV, a high thermal conductivity of >20 W/cm, and a high breakdown field of 10 MV/cm [1,2]. that make it suitable for high-power and high-frequency electronic applications. Moreover, diamond surface properties are also very attractive for bioelectronic devices due to their biocompatibility and good electronic performance. Nevertheless, these properties are very sensitive to chemical changes and, thus, the control and understanding of surface terminations are necessary for the implementation of diamond devices.

The most studied surface terminations of diamond are by far hydrogen-terminated (H-diamond) and oxygen-terminated (O-diamond) surfaces. The first is normally obtained by hydrogen plasma and shows a stable uniform 2 × 1 reconstruction, low roughness, a surface conductive layer, and negative electron affinity [3,4,5,6,7,8]. On the other hand, oxygen-terminated surfaces can be obtained by a wide range of treatments such as acid treatments [9,10], oxygen plasma [11], or vacuum ultraviolet (VUV)/ozone [9,12], among others. The resultant surface is not conductive and shows positive electron affinity. Additionally, O-diamond has been linked to a higher Schottky barrier height in metal/diamond contacts [13] in comparison to that based on H-diamond. 

Among the different diamond oxygenation treatments, acid treatment is one of the simplest and most used methods and has been extensively applied for the fabrication of diamond power devices. In contrast, VUV/ozone treatment leads to a more controlled process, improving the quality of the interfaces of the electronic devices [9,10,11,12]. A 1 × 1:O surface reconstruction has been deduced by surface electron diffraction-related techniques for different oxygenation treatments, including acid treatment [14,15,16]. The desorption of oxygen occurs in the form of CO molecules during an annealing process. The process occurs at a wide range of temperatures with a maximum desorption peak at about 600 °C [14]. After this, the diamond surface recovers a 2 × 1 reconstruction pattern that is the stable configuration in vacuum [4,5,6]. Concerning oxygen coverage, high similarities have been reported for the different oxidation treatments [17], even though some authors attribute a higher oxygen coverage for acid treatment in comparison to VUV/ozone treatment [9]. Among the C-O groups, bridge-bonded ether (C-O-C) and ketone (C=O) are the most reported candidates [9,18]. Although the stability of those surface configurations is still not clear, some calculations indicate that the hydroxyl and C-O-C states are the most favorable from an energy point of view [19]. Other results show that C-O-C is the main configuration at saturation coverages [18,20].

Diamond surface chemistry must be closely related to the type of hybridization of surface carbon and this, necessarily, to the nature of the bonded atom to carbon. While a H-diamond surface is usually linked to a C-C sp^3^ recovery, some experimental results point to sp^2^ carbon phases being generated during the oxidation process [18,21,22,23,24]. However, except for the ketone-based surface configuration [9], the rest of (100)-O-diamond surface reconstructions exhibit sp^3^ hybridized surface carbon atoms. Therefore, some degree of graphitization could be responsible for the observed sp^2^ phases. Although the diamond graphitization process has been extensively studied [23,25,26], its relationship with the oxidation process remains unclear. It is generally known that sp^2^-bonded carbon is faster oxygenated than sp^3^-bonded carbon and, indeed, some results linked the early stages of oxidation of diamond films to sp^2^-bonded carbon [27]. Moreover, surfaces generated by desorption of oxygen have shown more reactivity than surfaces generated by hydrogenated surface desorption [18,28] that can be also related to the remaining presence of sp^2^ carbon generated during the oxidation process.

X-ray photoelectron spectroscopy (XPS) has been widely used for analyzing diamond surface chemistry and electronic phenomena [29,30,31,32]. The presence of non-diamond C-C bond contributions in the C1s XPS spectrum has been widely reported in the case of O-diamond. This contribution appears at lower binding energies (BEs) than the diamond contribution. Its origin still remains under discussion but has been commonly linked to hydrocarbons adsorbed at the surface or graphitization. What seems to be usually observed is that this non-diamond carbon contribution is present with high reproducibility even for different oxygenation treatments [9,32,33,34]. However, this contribution is not revealed in H-diamond in which, ideally, all carbons are sp^3^ hybridized and the C-H bonds are related to higher BE values. On the other hand, most diamond XPS experiments are based on normal emission that, as detailed in Section 2, could not be sensitive enough to discriminate between the different surface bonding states. In this sense, the use of the angle-resolved mode of XPS allows to extrapolate with a high accuracy the different surface contributions. Unfortunately, such an approach is rarely used [29,34,35,36,37] and sometimes with a lack of quantification analysis.

In this work, the oxidation process of (100)-oriented diamond surface is investigated by angle-resolved XPS (ARXPS) and compared to a H-diamond. Previously, this (100)-oriented diamond was treated by hydrogen plasma to have the same initial surface state as starting point in both samples, so that the surface bonding evolution during the O-treatment could be evidenced. The XPS peak attributions of the ARXPS for the H-diamond were previously discussed in detail in [29]. Analysis is here focused on the explanation of the non-diamond C1s contribution in O-diamond. Based on the ARXPS results, a novel 1 × 1:O reconstruction model for the (100)-O-diamond is proposed.

## 2. Materials and Methods

For this experiment, a high-pressure high-temperature (HPHT) 3 × 3 mm^2^ diamond (100)-oriented type IIa substrate was used. Before H_2_ plasma treatment, the sample was cleaned by a hot acid mixture HClO_4_:H_2_SO_4_:HNO_3_ (1:3:4) for 2 h. The temperature of the heating platform was adjusted to 450 °C. Then, the sample was consecutively submerged in an acetone, ethanol, and isopropanol ultrasound bath for 5 min each. The substrate was then dried by Ar gas flow. H_2_ plasma was performed in a NIRIM-type reactor using a microwave power of 260 W, at a pressure of 30 Torr, a gas H_2_ flow of 200 sccm, and a temperature of ~800 °C for 2 h. The ARXPS analysis of the resultant H-diamond surface has been published elsewhere [29]. Finally, the H-diamond sample was submitted again to a final hot acid mixture to obtain the O-diamond surface.

Surface morphology was evaluated by atomic force microscopy (AFM) in a VEECO NSIV system (VEECO, New York, NY, USA) working in tapping mode. Gwyddion software (version 2.52) was used for AFM data analysis [38]. AFM measurements showed an increasing roughness from *Rq* ~ 0.12 nm and *Ra* ~ 0.09 nm (after the H_2_ plasma) to *Rq* ~ 0.3 nm and *Ra* ~ 0.12 nm (after acid treatment).

**Figure 1:**
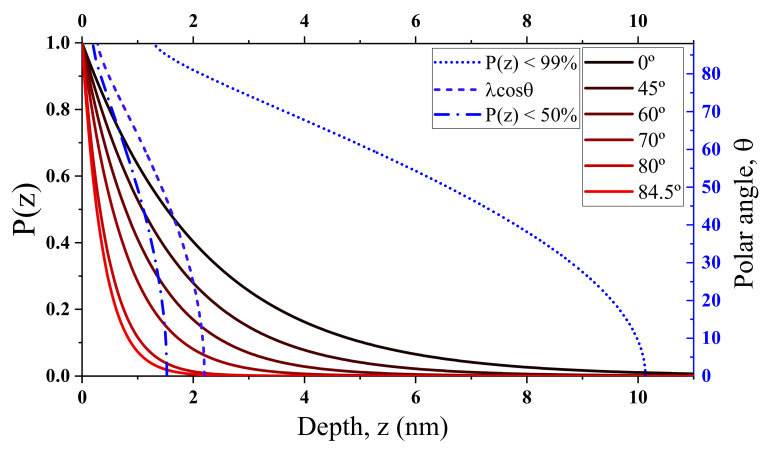
Depth sensitivity estimation of angle-resolved X-ray photoelectron spectroscopy (ARXPS) measurements in diamond for the present work conditions. The graph shows the electron escape probability P(z) of C1s photoelectrons (left Y-axis) versus depth for different polar angles (solid lines) and the P(z) < 99%, P(z) < 50%, and *λcosθ* depth curves (discontinued lines) versus polar angles (right Y-axis). P(z) < 99% is an estimation of the maximum depth sensitivity, while P(z) < 50% represents the depth from which half of the total signal is generated. The *λcosθ* value is useful to extract a rapid approximation of the depth sensitivity. The refraction effect is taken into account which becomes not negligible when the polar angle increases.

ARXPS spectra were carried out in a Kratos Axis Ultra DLD spectrometer (Kratos analytical, Manchester, UK) at ultra-high vacuum and room temperature with an Al k-α radiation source (1486.6 eV) with an accuracy of 0.1 eV and an energy pass of 20 eV. The spectra were calibrated versus the Au4f_7/2_ peak located at 84.0 eV and were recorded for electron polar angles of θ = 0°, 60°, 70°, 80°, and 84.6° to the surface normal so that the higher the polar angle the more surface sensitive the measurement is (see Figure 1). In this system, the stage needs to be tilted to achieve the required polar angle. The angle between the X-ray beam and the detection column remains constant. A charge neutralizer based on a flood gun of low-energy electrons was used to avoid charge effects.

To give an idea of the depth sensitivity of the different polar angle spectra, the electron escape probability P(z) is represented in Figure 1 (solid lines). P(z) is deduced using by the Beer–Lambert law P(z) *= e^−z/λcosθ^*, where *z* is the depth from surface (*z* = 0), *λ* is the attenuation length and *θ* is the polar angle. The attenuation length has been previously estimated experimentally as 2.4 nm for diamond [39]. 

The curves shown in Figure 1 take into account the refraction effect and, thus, the corresponding effective polar angles are 0° (for *θ* = 0°), 44.57° (for *θ* = 45°), 59.26° (for *θ* = 60°), 68.85° (for *θ* = 70°), 77.80° (for *θ* = 80°), and 80° (for *θ* = 84.6°). The maximum depth sensitivity can be well represented by P(z) < 99% (Figure 1 (dotted line)). Its value is 10.1 nm and 1.8 nm for 0° and 84.6° polar angles, respectively. However, since P(z) has an exponential behavior, the collected electrons are more representative of the first nanometers than for the deepest ones. To illustrate this, the P(z) < 50% is also shown in Figure 1 (dashed line). Note that it represent the depth from which half of the XPS signal can get away from the surface. Its values for 0° and 84.6° polar angles are 1.22 nm and 0.26 nm, respectively. The *λcosθ* value (Figure 1 (dashed-dotted line)) has been commonly used to have a rapid approximation to the depth sensitivity.

The XPS background has been subtracted using the Tougaard model [40]. Peak deconvolution was carried out using OriginPro software (version 8, OriginLab corporation, Northampton, MA, USA). The deconvolution parameters are detailed in Section 3.2.

## 3. Results and Discussion

### 3.1. C1s Spectra Comparison

**Figure 2:**
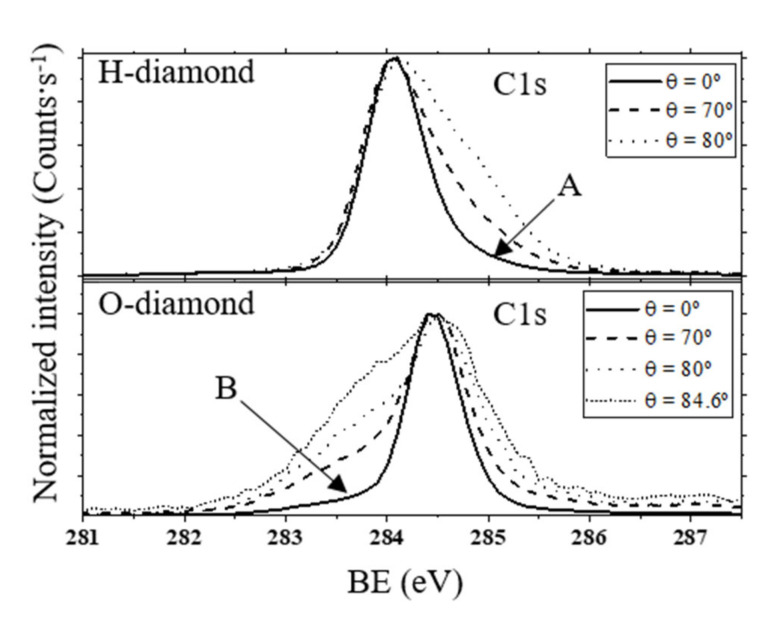
Normalized ARXPS C1s spectra for H-terminated diamond (H-diamond) (top) and O-terminated diamond (O-diamond) (bottom) surfaces at different polar angles. The position of the maximum C1s peak is shifted ~0.4 eV as widely reported in the literature. Surface contributions at higher BEs are observable for higher polar angles in H-diamond. In contrast, O-diamond shows surface contributions at both sides of the main peak. The contribution at lower BEs is related to non-diamond C-C contributions. This component is not present in H-diamond.

The ARXPS C1s spectra for the H-diamond and O-diamond (100) surfaces are compared in Figure 2. First, the diamond bulk position and width can be estimated from the 0° spectrum as, in that case, most of the signal is coming from up to ~10 nm deep as estimated in Section 2 and, thus, diamond bulk must be the main contribution. The position of diamond sp^3^ carbon peaks is located at ~284.05 eV and ~284.45 eV for H-diamond and O-diamond, respectively. This energy difference of ~0.4 eV between H- and O-diamond has been widely reported, but the reasons for this effect still remain unknown. In [32], this C1s maximum peak shift was related either to an upward band bending in H-diamond (related to the surface transfer doping phenomenon [41]) and to a downward band bending in O-diamond. In both cases, the band bending should be defined deeper than the XPS depth sensitivity. In any case, the position of the diamond sp^3^ peak is very reproducible even using different experimental setups and procedures [9]. In this sense, the C1s spectra reported in the literature, most of them at 0° polar angle, were compared. The asymmetric 0° C1s spectrum with a tail towards higher BEs (Figure 2, arrow A) is also widely reported for (100)-H-diamond [34], while the opposite tail, towards lower BEs (Figure 2, arrow B), is reported in the case of most of (100)-O-diamond XPS studies, even for those treated by VUV/ozone [9]. The reason for these tails is, in fact, the presence of surface contributions (Figure 2). At 0° polar angle, these surface contributions have a very low intensity in comparison to the bulk contribution, which is only intense enough to transform the bulk peak into an apparent asymmetric shape.

Surface contributions are clarified by the observation at higher polar angle XPS spectra (Figure 2). The increasing intensity of contributions along the tail energy positions (arrows A and B) is observed. These new contributions are necessarily related to additional superficial contributions than the diamond sp^3^ bulk one. The evolution of these surface-related contributions is very different between H-diamond and O-diamond, which evidenced a totally different surface bonding configuration. All surface components are placed towards higher BEs in the H-diamond surface. Concerning the O-diamond, this shows a big contribution towards lower BEs and a smaller one at higher BEs. For the latter, C-O contributions are the main candidates. The contributions with BEs lower than diamond sp^3^ carbon could be related to non-diamond carbons with different chemical environments related to defects or sp^2^ hybridizations, contamination, surface band bending, and carbides. The latter has been discarded since no other elements as C and O are detected in the survey spectra. Concerning surface band bending, there is no evidence that a charge is generated at the very near surface as in the case of H-diamond [29,42]. The presence of defects or contamination could be considered, but the high reproducibility of the peak at different experimental procedures, for example, in the use of chemical vapour deposition ([34]) or HPHT (this work) substrates, together with the absence of this peak for the H-diamond fabricated from the same substrate, lead one to think that this contribution exists as a consequence of the oxygenation treatment. Thus, non-diamond carbon contributions related to the presence of sp^2^ hybridization bonds are the most reliable explanation. Some results also evidenced the partial sp^2^ carbon formation at diamond surface during acid treatments, based on optical properties [9], Raman spectroscopy [18,21], and electron loss spectroscopy [18] experiments.

### 3.2. O-Terminated Diamond C1s Spectra Deconvolution

**Figure 3:**
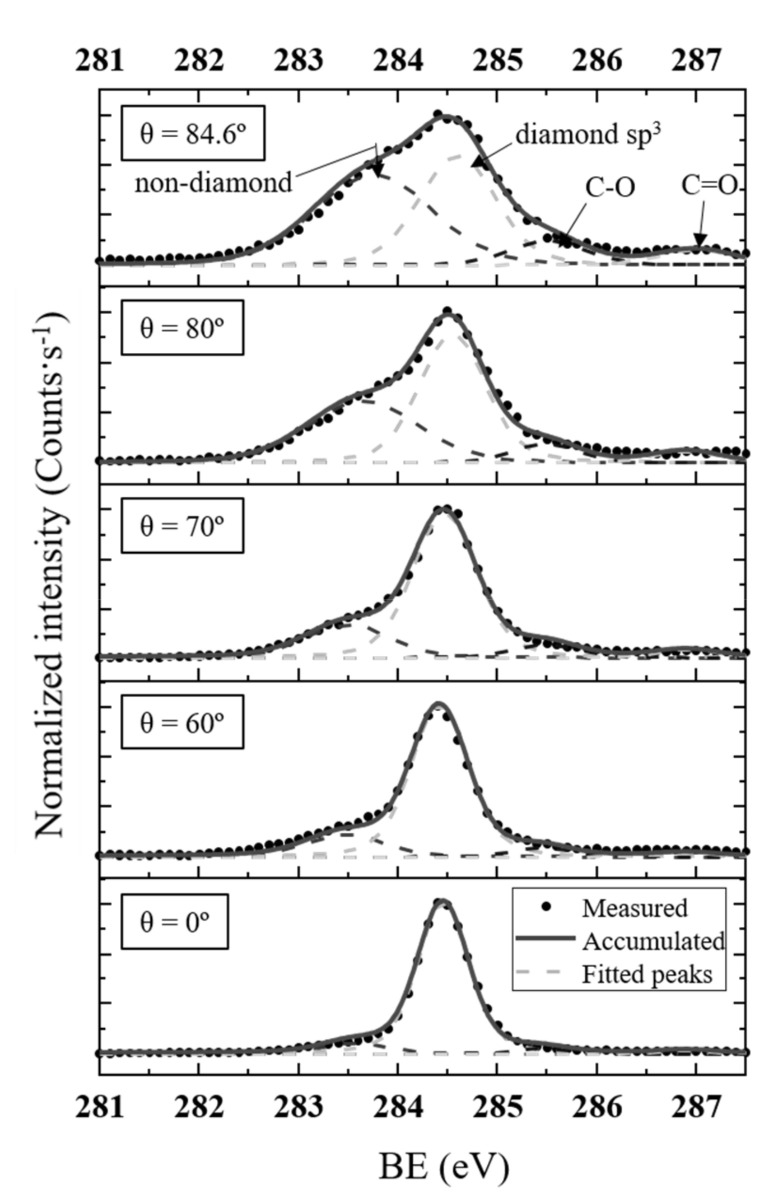
Normalized ARXPS C1s spectra deconvolution of O-diamond for different polar angles. Four peaks were considered in the deconvolution. From lower to higher BEs: non-diamond C-C bonds, diamond sp^3^, C-O (simple bond groups such as hydroxyl or C-O-C bridges), and C=O (double bond groups such as ketone). The diamond peak is placed at 285.45 eV. The Gaussian width of the non-diamond peak is wider possibly because it is formed as the sum of various peaks related to diverse carbon chemical environments (hybridization, defects, etc.). The C-O and C=O areas matched well with the O1s contribution.

Deconvolution of the C1s spectra of the O-diamond surface and their evolution with angle is shown in Figure 3. A similar procedure has been carried out and published elsewhere for the H-diamond [29]. Following the peak attribution previously explained in Section 3.1, four peaks have been used for the spectra deconvolution: sp^3^ carbon (diamond); non-diamond carbon (related to sp^2^ and defects); C-O simple bonds such as C-O-C bridges or hydroxyl (C-OH) groups, whose energy position should be around 285.5–286.5 eV; and C=O double bonds such as ketones with positions over 286.5 eV. The proposed simple bond C-O groups were reported to be the most energetically favorable for (100) diamond [19]. The position of oxygen groups was set according to previous XPS studies [9,15,34,43]. In principle, it is difficult to discern if the oxygen groups are related to sp^3^ or sp^2^ carbon groups because the peak positions are analogous to that of graphite and graphite oxide [44]. Moreover, similar non-diamond contributions are widely reported up to the present in XPS O-diamond and, thus, the C-O contributions for diamond can generally be linked to sp^2^ carbon phases. The position of the non-diamond carbon peak is ~283.5 eV. The energy distance between the diamond and the non-diamond contribution peak observed here agrees well with previously published theoretical works related to sp^2^ and sp^3^ carbon contributions [45].

Regarding XPS peak shapes, the convolution of Gaussian and Lorentzian, known as Voigt distribution, are extensively used. The Lorentzian width was fixed to 0.2 eV based on the graphite lifetime broadening result [46], and the Gaussian width is dependent on the spectrometer energy resolution and, in turn, on the polar angle. The dispersive monochromatic Al *k*-α light is incident on the sample and the electron lens system takes photoelectrons from the different size area of the sample depending on the polar angle. This makes the energy resolution change and hence the Gaussian width of contributions is expected to increase with polar angle. In this sense, it was set for each polar angle using the Au4f_7/2_ peak as reference. The Gaussian width value for *θ* = 0° for diamond peak was set to 0.5. For the non-diamond carbon peak, the Gaussian width is higher than that of diamond contribution, which, in principle, could be related to the presence of several carbon chemical environments. Thus, all these contributions are here integrated under the same “non-diamond” peak label. For this peak, the Gaussian width was set to follow an analogous width trend to that of the diamond peak.

On the other hand, the total area of oxygen contributions in the deconvoluted C1s spectra has been compared to the O1s area as if all detected oxygen is attributed to surface C-O and C=O bonds. The relative sensitivity factors are then applied to the area of the O1s peak for each polar angle measurement. Thus, the total area of oxygen contributions in the deconvoluted C1s spectra matches well to the corresponding area estimated from O1s spectra. This supports the conclusion that most of the O1s contribution should be related to C-O and C=O bonds. A more detailed analysis of O1s spectra is shown in Section 3.3.

Neither C-O nor non-diamond carbon contributions here revealed were previously evidenced in H-diamond [29]. In addition to the diamond contribution, the H-diamond deconvolution was based on C-H bonds and on the presence of a surface downward band bending [29,42]. The presence of a non-diamond carbon XPS component was also detected in surfaces obtained by other oxygenation treatments such as VUV/ozone-treated surfaces at different pressure conditions [9]. Small amounts of sp^2^ carbon have been also detected by other techniques [18,21,47]. In [47], sp^2^ carbon presence was evidenced at the surface by X-ray absorption spectroscopy for acid-treated O-diamond. This sp^2^ component was not detectable for H-diamond. ARXPS experiments carried out on acid-treated boron-doped (100)-diamond surface were recently reported [34]. Their spectra exhibit the same four contributions described in Figure 3 with the same attributions. The component attributed to non-diamond carbon structures in this work was attributed in [34] to extrinsic hydrocarbon molecules adsorbed on the surface. Other studies pointed out that diamond oxidation is accompanied by surface graphitization of diamonds [22] without a considerable increase in the overall oxygen content [17]. Thus, surface graphitization was observed in diamond at temperatures much lower than those required for “true” graphitization and was believed to result from oxidation [18,23].

The resultant deconvolution leads one to think that the diamond oxygenation process is linked to the formation of non-diamond carbon structures on the very near-surface region, which, at least partially, must be related to the C-O bonds detected by XPS. Present results are in agreement with the bond configuration generally reported in the literature for the O-diamond surface bond configuration. Based on this deconvolution, a sample modelling, using different layer-related XPS intensities, is proposed in Section 3.4 to quantify the non-diamond carbon contributions as well as the oxidized layers.

### 3.3. O1s Spectra Comparison

Concerning O1s spectra, it must be noted again that most of the oxygen quantification in the literature has been made by using the 0° XPS spectrum. At this angle, the C1s XPS signal comes from the first ~10 nm and most of the signal is related to diamond sp^3^ contribution. This peak is then very weak and, thus, atomic %O estimated from the 0° polar angle (according to Equation (1)) is not very accurate and does not give any representative information on the real oxygen coverage. Using different polar angles, variations of the different peaks can be observed, as performed in the C1s spectra, and information from the surface-related contributions can be deduced. Moreover, the higher the polar angles the lower the depth sensitivity is and, thus, the estimated atomic %O should be more accurate and representative of the surface coverage.

**Figure 4:**
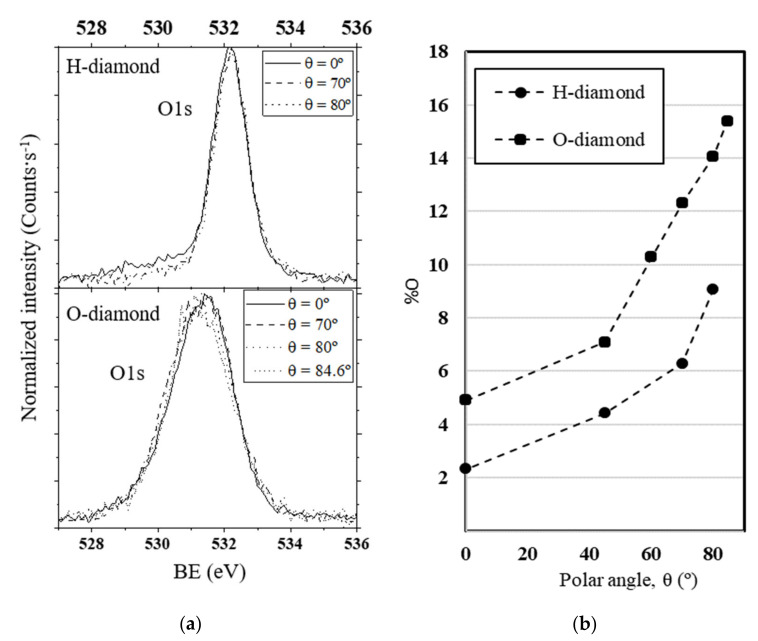
ARXPS oxygen data comparison for H- and O-diamond showing the following: (**a**) normalized O1s ARXPS spectra—no qualitative variations are observed along with the sample depth, O-diamond spectra show a wider O1s peak; and (**b**) atomic %O obtained from Equation (1)—the O1s contribution is higher for O-diamond at every polar angle. The higher O1s width and intensity in O-diamond are related to the formation of surface C-O and C=O groups. The quantification error is estimated as 5–10%.

Normalized ARXPS O1s spectra at different polar angles for H- and O-diamond are represented in Figure 4a. In both cases, the O1s peak shapes remain mostly invariable for different polar angles, which means that there are no qualitative variations along with the sample depth. It is worth noting that the width of the O1s peak in H-diamond is nearly half that of O-diamond. This enlargement of the O1s peak is attributed to the formation of non-diamond carbon structures at the surface in O-diamond that should be related to C-O contributions at lower BEs. The appearance of this new contribution also causes the O1s peak to shift towards lower BEs. Moreover, the C1s sp^3^ to O1s energy distance in H-diamond is very similar to that of the C1s non-diamond to O1s in O-diamond. From this observation, it is suspected that a link between the new O1s component and the non-diamond C1s peak in O-diamond exists. In this sense, neither non-diamond carbon nor clear C-O contributions were detected in H-diamond (see Figure 2).

In order to quantitatively compare the oxygen contents of H- and O-diamond, the atomic %O at each polar angle were deduced using the following equation:

(1) %O=IO1sRSFO1sIC1sRSFC1s+IO1sRSFO1s·100

where *I_C1s_* and *I_O1s_* are the peak intensities for *C*1*s* and *O*1*s* spectra, respectively, and *RSF_C_*_1*s*_ and *RSF_O_*_1*s*_ are the relative sensitivity factors provided by the XPS manufacturer for *C*1*s* and *O*1*s*, respectively. The results are summarized in Figure 4b. The quantification error is estimated as 5–10%. It must be noted that, based on Equation (1), a total coverage is related to a certain %O value, which indeed depends on a model of the sample surface. In any case, a higher %O for all the polar angles is observed on the O-diamond. The value deduced for *θ* = 80°, related to the first 2 nm of the sample, is the most surface representative and, in that region, the %O for O-diamond is 1.55 times that of H-diamond. However, some caution must be used when comparing oxygen contents since these coverages can include partially adsorbed water molecules [48,49]. Furthermore, previous XPS experiments show that oxygen coverage decreases after several days in vacuum, which is attributed to the desorption of water molecules [34]. In such cases, the water contribution can hardly be resolved from that of oxygen atoms chemically bonded on diamond surface by observing the *O*1*s* spectrum. Thus, only the observation of C1s can give some information about the real C-O contributions. From H-diamond C1s spectra (see Figure 2), the absence of contributions in the C-O region even at a high polar angle leads one to think that most of the oxygen detected in H-diamond was not chemically bonded to carbon. This idea is in agreement with the water overlayer model and the surface conductivity mechanism proposed in [41]. Conversely, these C-O contributions are clear in *C*1*s* spectra for O-diamond and, as commented in Section 3.1, there intensities are in good agreement with the *O*1*s* intensity. For this reason, we conclude that, while a partial water contribution cannot be discarded, most of the detected oxygen in O-diamond is chemically bonded to carbon atoms.

### 3.4. Surface Model and Quantification

The XPS peak intensities are sensitive to the quantity of material emitting the XPS electrons and to the distance to the free surface. The relative intensity of each peak recorded during the XPS experiments follows this principle. Here, three types of C1s contributions are recorded: diamond sp^3^ carbon atoms at 284.45 eV, carbon atoms bonded to oxygen (C-O and C=O, at 285.5 and 287 eV, respectively), and, finally, carbon atoms that are bonded to other carbon atoms but with chemical environment modified in terms of hybridization, chemical structure, or presence of defects at 283.5 eV. The integration of those peaks gives relative intensities proportional to the number of each type of chemical environment and its distance to the surface.

Using these relative intensities, one can deduce the resulting bonding configuration after an oxygenation process using a three-layer model. The choice in dividing the diamond surface region into three layers comes from the observations that indicate that the acid treatment modifies subsurface in terms of graphitization. The non-diamond and the C-O contributions are placed over the diamond contribution as deduced from the ARXPS spectra peak evolution (see Figure 3). Indeed, oxygen should be at the surface, the observed non-diamond carbon immediately below, and diamond sp^3^ in the bulk region.

**Figure 5:**
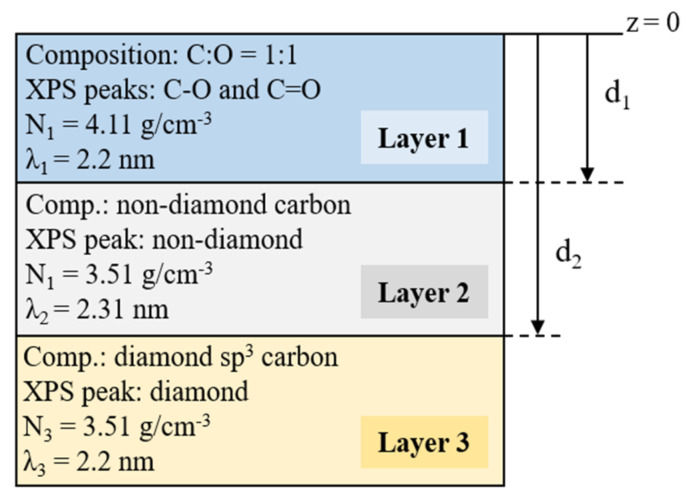
Schematic of the sample model and its representative parameters: composition, attributed XPS peak, density, attenuation length and thickness. The sample is divided into three layers: Layer 1, C-O bond layer; Layer 2, non-diamond carbon-containing layer; and Layer 3, diamond bulk. The density of Layers 2 and 3 was set to diamond values, *N*_2_ = *N*_3_ = 3.51 g/cm^3^. The density of Layer 1 was calculated using the atomic density of diamond, a C:O = 1:1 ratio, and the respective molar mass values for C and O. The attenuation length for Layer 2 was set to graphite value, while for Layers 1 and 3 it was set to diamond value. Finally, *d*_1_ and *d*_2_ correspond to the thicknesses of Layers 1 and 2, respectively.

Thus, we propose to consider three homogeneous layers: (i) Layer 1, a superficial C-O bonding layer; (ii) Layer 2, a subsurface layer consisting of the partial modification of the diamond sp^3^ bonding resulting from the acid treatment, corresponding to the non-diamond peak intensity; and (iii) Layer 3, the bulk diamond, corresponding to the diamond sp^3^ XPS peak. Figure 5 gives a schematic description of the layer configuration proposed for this model. These layers correspond to C1s XPS contributions as follows: Layer 1, C-O and C=O peaks; Layer 2, non-diamond peak; and Layer 3, diamond sp^3^ peak. Homogeneous and atomically flat layers neglecting surface roughness and inhomogeneity effects are assumed. The effects of electron refraction have been considered. Thus, the same procedure as in [29] has been used. For an extensive discussion of these effects and the validity of the above assumptions, see [50].

The thicknesses of Layers 1 and 2 can be estimated from any of the C1s peak intensity ratios as widely used for metal-oxide thickness estimation [51]. In this work, the peak intensity ratios I_1_/I_2_ and I_2_/I_3_ are estimated as follows:

(2) $$\frac{I_{1}}{I_{2}}=\frac{N_{1}\lambda_{1}}{N_{2}\lambda_{2}}\cdot \frac{1-\exp\left(\frac{-d_{1}}{\lambda_{1}cos\theta }\right)}{1-\exp\left(\frac{-d_{2}}{\lambda_{2}cos\theta }\right)\cdot \exp\left(\frac{-d_{1}}{\lambda_{1}cos\theta }\right)}$$

(3) $$\frac{I_{2}}{I_{3}}=\frac{N_{2}\lambda_{2}}{N_{3}\lambda_{3}}\cdot \frac{1-\exp\left(\frac{-d_{2}}{\lambda_{2}cos\theta }\right)}{\exp\left(\frac{-d_{2}}{\lambda_{2}cos\theta }\right)}$$

where *I*, *N*, *d*, and *λ* refer to the XPS intensity, density, thickness, and attenuation length of a layer, respectively. The subscripts indicate the layers to which these properties are attributed. The density for Layers 2 and 3 was set to diamond values, that is *N*_2_ = *N*_3_ = 3.51 g/cm^3^. The attenuation length for Layers 1 and 3 was set to diamond value *λ*_1_ = *λ*_3_ = 2.2 nm, while Layer 2 was set to graphite value *λ*_2_ = 2.31 nm. In this sense, the attenuation length of graphite is known to be very close to diamond despite the big density difference and has been estimated using the expression *λ*_graphite_ = *λ*_diamond_/0.95 = 2.31 nm [52,53]. For Layer 1, the density was obtained by considering the molar mass of oxygen and carbon and the atomic density of diamond, supposing that the C-O layer composition is C:O = 1:1. A summary of the model parameters is shown in Figure 5.

**Figure 6:**
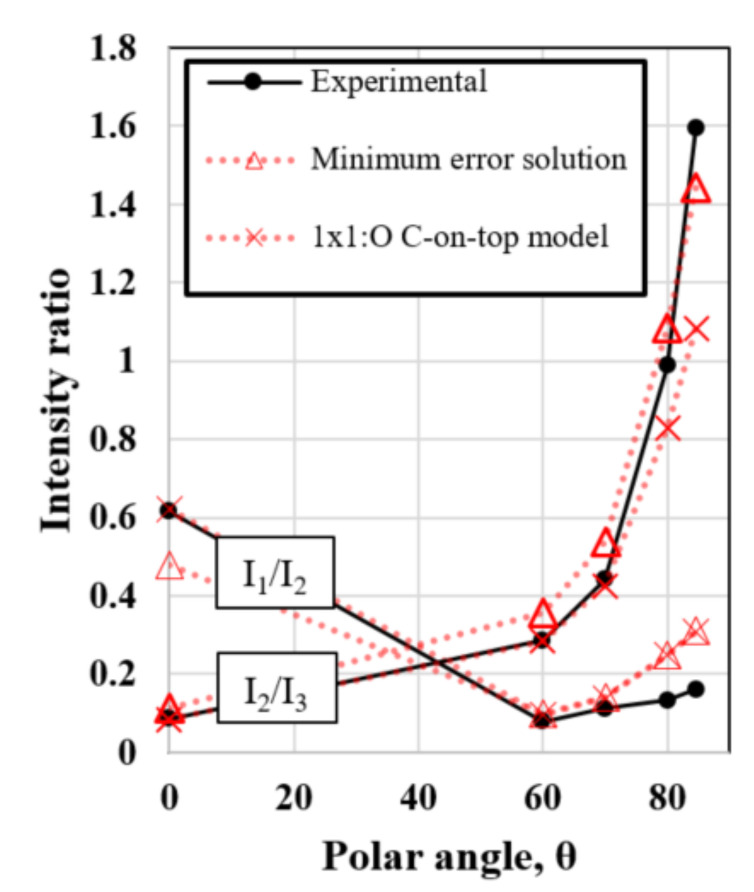
Graph showing the intensity ratios I_1_/I_2_ and I_2_/I_3_ obtained experimentally from XPS peak areas (solid line) and obtained by applying Equations (2) and (3) (dotted lines) at different polar angles. Two solutions are presented: the minimum error solution (*d*_1_ = 0.089 nm and *d*_2_ = 0.237 nm) and the (100) 1 × 1:O “C-on-top” model (*d*_1_ = 0.089 nm and *d_2_* = 0.178 nm). The error is calculated as the sum of the errors of both *I*_1_*/I*_2_ and *I*_2_*/I*_3_ estimators at every polar angle.

**Figure 7:**
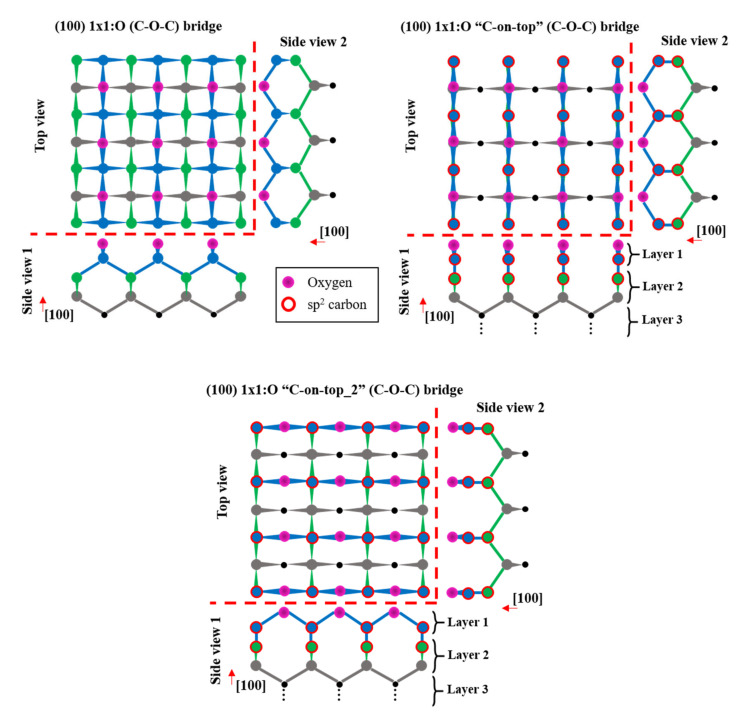
Schematic image (top and side views) of the four topmost layers of carbon atoms and the surface oxygen atom layer of the (100) diamond surface. The oxygen atoms are in purple and the sp^2^ carbon is marked by a red circumference. Top-Left side: the (100) 1 × 1:O (C-O-C) bridge reconstructed surface is based on full sp^3^ hybridized carbon. The carbon atoms of the first layer are bonded to two surface oxygen atoms and two carbon atoms in the second layer. Top-Right and Bottom side: the (100) 1 × 1:O “C-on-top” and “C-on-top_2” reconstructions, respectively, are proposed in this work. In both cases, the carbon atoms of the first layer are bonded to two surface oxygen atoms and one carbon layer of the second layer and are positioned just on top of them. Thus, the first and second carbon atom layers are sp^2^ hybridized.

*I*_1_*/I*_2_ and *I*_2_*/I*_3_ were then obtained experimentally from XPS peak intensities. Then, the values of *d_1_* and *d_2_* were optimized to reduce the sum of the errors of the estimators (2) and (3) at every polar angle. The optimal solution occurs for the thickness values *d*_1_ = 0.089 nm and *d*_2_ = 0.237 nm. The experimental and theoretical values for *I*_1_*/I*_2_ and *I*_2_*/I*_3_ are presented in Figure 6. As can be seen, the proposed three-layer model fits well the experimental results. The value of *d_1_* corresponds to 1 monolayer (ML), which is defined as the distance between two consecutive diamond (100) planes. It means full coverage of C-O and C=O bonds. The combination of non-diamond and diamond carbon contributions into Layer 2 was also considered, but the solutions were not optimal. Thus, this solution supports the conclusion that the carbon atoms just below the oxygenated layer are in a different chemical environment than that of diamond. Furthermore, the very low thickness of this subsurface carbon layer (*d_2_* = 0.237 nm) and the high reproducibility of this contribution in XPS lead one to think about whether the XPS spectra can be generated by a reconstruction of the surface that includes the presence of carbon atoms in a non-diamond structure. In this sense, most of the proposed (100) 1 × 1:O reconstruction models in the literature are based on a full sp^3^ hybridized carbon structure. One of the most accepted 1 × 1:O models is based on C-O-C bridges [18,20] (see Figure 7 (Top-Left side)). However, this as well as other possible reconstructions based on sp^3^ carbon [54] are not compatible with the experimental evidence of non-diamond carbon contributions. Other possible reconstructions are based on ketone groups, in which the top-layer atoms are sp^2^ hybridized. In this case, ketone groups can be easily detected in XPS and are known to not be the dominant surface C-O group. Thus, the presence of non-diamond carbon subsurface contribution cannot be fully justified by ketone groups.

A different reconstruction model could explain the presence of the subsurface non-diamond carbon layer as well as being compatible with previous experimental results. In Figure 7 (Top-Right side), a schematic of the proposed (100) 1 × 1:O “C-on-top” and “C-on-top_2” models are represented. In both reconstructions, the carbon atoms of the first and second layers are sp^2^ hybridized. The carbon atoms of the first layer are placed just on top of the atoms of the second layer and are bonded to them. The carbon atoms of the third layer are sp^3^ hybridized but are bonded to two sp^2^ carbons of the second layer and two sp^3^ carbons of the fourth layer. Therefore, the atoms of the second and third layers have different chemical environments than in a pure diamond structure due to the presence of sp^2^ carbons. Thus, its contributions in C1s XPS spectra should be at lower BEs than diamond and will lead to two different peaks. These facts would justify the presence of the non-diamond carbon XPS contribution and its higher peak width. Additionally, the carbon atoms of the first layer correspond to the C-O XPS contribution at ~285.5 eV. The correspondence of the layers used in the quantification model with the atomic layers of the reconstruction model is shown in Figure 7.

On the other hand, the partial coverage by ketone groups is also compatible with the present XPS result. In ketone groups, the carbon atoms bonded to oxygen are related to the contribution at 287 eV and also contribute to Layer 1. These carbon atoms are sp^2^ hybridized and, for this reason, the carbon atoms of the second layer are in a different chemical environment than those in diamond and should be related to lower BE peaks. The contribution of the second atomic layer in the ketone-based reconstruction would be also integrated into the non-diamond XPS peak, and therefore, is related to Layer 2 of the quantification model.

Additionally, the parameters used in the quantification model should not differ from those of the proposed reconstruction model. As already commented, the attenuation length values for diamond and graphite are very similar (2.2 and 2.31 nm, respectively). Thus, the presence of sp^2^ carbon would hardly change the attenuation length of Layer 2 and, therefore, neither would the estimated result. Concerning the density values, it must be noted that the areal atomic densities of the topmost atomic layers in the proposed reconstruction are the same as those in the ideal diamond structure since it can be virtually obtained by an in-plane lateral shift of the complete first atomic layer. Thus, only the volumetric density modification is expected as a result of the different bonding structure, which would change the distance between (100) atomic planes of the topmost layers. 

The theoretical estimations of the two versions of the (100) 1 × 1:O “C-on-top” reconstruction to the XPS result are the same and have been included in Figure 6. It must be noted that these models are considering the partial covering of ketone groups. The thicknesses of Layers 1 and 2 were fixed to 1 ML and 2 ML, respectively. Due to the different carbon bonding structure in Layer 2, the (100) interplanar distance should differ from the ideal 2 ML of the diamond structure. As noted, the models fit well the experimental values. The error increases for the higher polar angle data, which can be related to other surface phenomena whose effects were not considered here, such as roughness. It must be noted that the proposed 1 × 1:O models up to today are not compatible with the XPS results presented here because the non-diamond carbon states remain unexplained. Further calculations concerning the formation mechanisms and stability of the models exposed here are still in progress.

## 4. Conclusions

In this work, the chemistry of the O-diamond surface was analyzed by ARXPS and compared to H-diamond showing the chemical changes occurring during the acid treatment. The results lead one to conclude that the effect of the acid treatment on the H-diamond is dual, namely the oxygenation of the surface and the formation of a subsurface non-diamond carbon layer. The existence of C-O and non-diamond carbon contributions together with a ~0.4 eV shift towards higher BEs of the diamond sp^3^ contribution define the transition from H- to O-diamond surface in the C1s XPS spectra. From O1s, the atomic %O is higher for O-diamond. In contrast to H-diamond, most of the O1s contribution comes from oxygen bonded to carbon. A three-layer sample model was used for O-diamond surface quantification. Oxygen coverage of 1 ML and a subsurface non-diamond carbon layer of 2.67 ML were estimated. To explain this result, a novel (100) 1 × 1:O reconstruction model was proposed. In contrast to most of the previous models, this model includes non-diamond carbon structures. Further calculations concerning the formation mechanism and stability of the proposed reconstruction are now in progress and will be presented in future work.
